# Supplementary material for: The Construction and Exploration of a Comprehensive MicroRNA Centered Regulatory Network in Foxtail Millet (Setaria italica L.)
Source: Front Plant Sci. 2022 May 6;13:848474. doi: 10.3389/fpls.2022.848474 (PMC9121102; doi:10.3389/fpls.2022.848474)
Supplement: Supplementary file 4 [file Data_Sheet_4.zip › Sit-miR172a_Seita.4G010900.1_1622_TPlot.pdf]

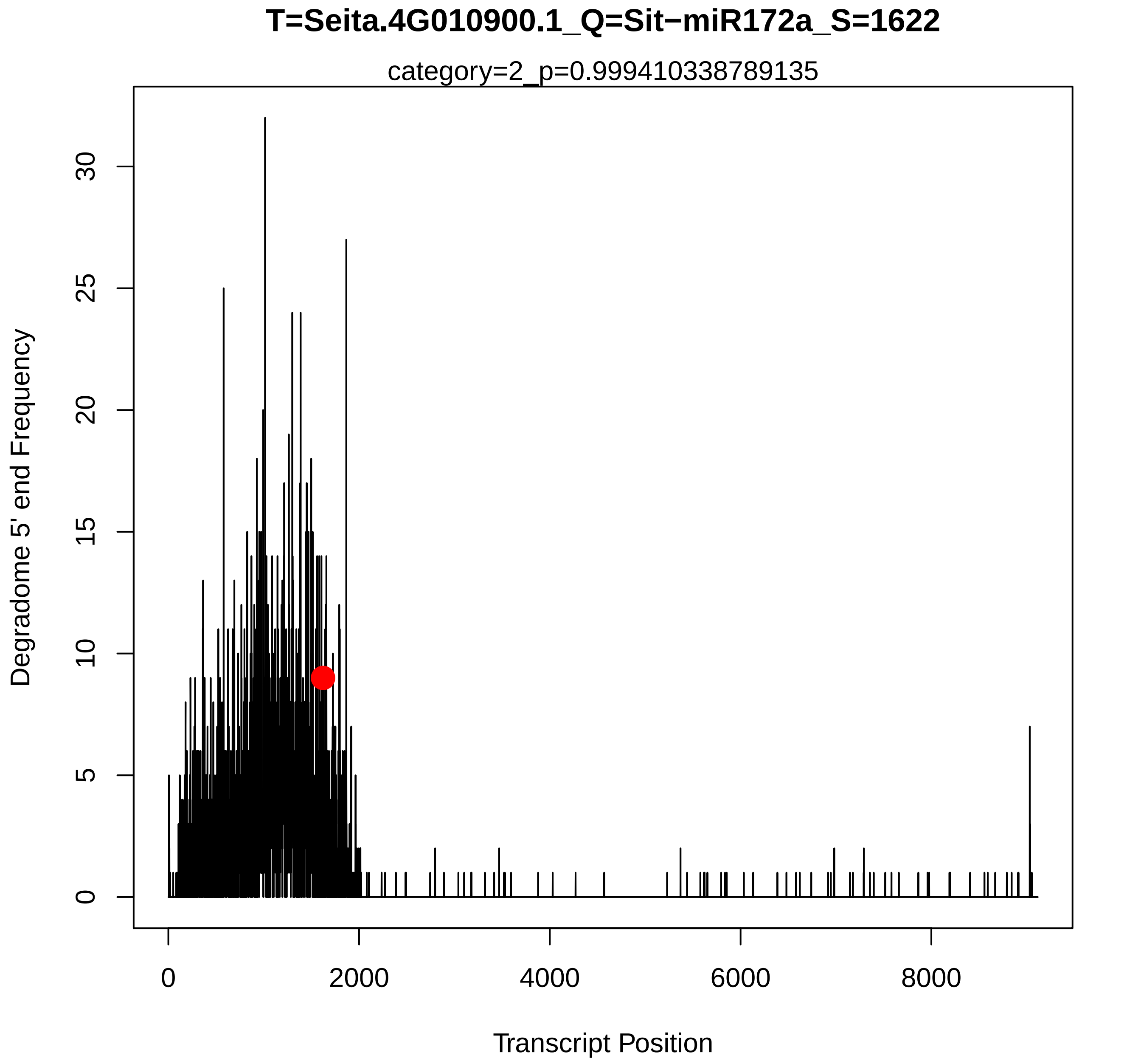
T=Seita.4G010900.1_Q=Sit−miR172a_S=1622
category=2_p=0.999410338789135
Degradome 5' end Frequency
0
5
10
15
20
25
30
0
2000
4000
6000
8000
Transcript Position
